# Inhibitory Effect of Etravirine, a Non-Nucleoside Reverse Transcriptase Inhibitor, via Anterior Gradient Protein 2 Homolog Degradation against Ovarian Cancer Metastasis

**DOI:** 10.3390/ijms23020944

**Published:** 2022-01-15

**Authors:** Thanh Truong Giang Ly, Jisoo Yun, Jong-Seong Ha, Yeon-Ju Kim, Woong-Bi Jang, Thi Hong Van Le, Vinoth Kumar Rethineswaran, Jaewoo Choi, Jae-Ho Kim, Sang-Hyun Min, Dong-Hyung Lee, Ju-Seok Yang, Joo-Seop Chung, Sang-Mo Kwon

**Affiliations:** 1 Laboratory for Vascular Medicine and Stem Cell Biology, Department of Physiology, Medical Research Institute, School of Medicine, Pusan National University, Yangsan 50612, Korea; lythanhtruonggiang@gmail.com (T.T.G.L.); jsyun14@hanmail.net (J.Y.); jongseong@pusan.ac.kr (J.-S.H.); twou1234@nate.com (Y.-J.K.); jangwoongbi@naver.com (W.-B.J.); lethihongvan25121978@gmail.com (T.H.V.L.); vinrebha@gmail.com (V.K.R.); wozh1304@naver.com (J.C.); 2 Convergence Stem Cell Research Center, Pusan National University, Yangsan 50612, Korea; jhkimst@pusan.ac.kr; 3 New Drug Development Center, Deagu Gyeongbuk Medical Innovation Foundation, Deagu 41061, Korea; shmin03@dgmif.re.kr; 4 Department of Obstetrics and Gynecology, Pusan National University Yangsan Hospital, Yangsan 50612, Korea; ldh0707@hanmail.net (D.-H.L.); yangandshin@gmail.com (J.-S.Y.); 5 Department of Hematology-Oncology, Pusan National University Hospital Medical Research Institute, Busan 49241, Korea

**Keywords:** AGR2, etravirine, autophagy, ovarian cancer

## Abstract

Anterior gradient protein 2 homolog (AGR2), an endoplasmic reticulum protein, is secreted in the tumor microenvironment. AGR2 is a member of the disulfide isomerase family, is highly expressed in multiple cancers, and promotes cancer metastasis. In this study, we found that etravirine, which is a non-nucleoside reverse transcriptase inhibitor, could induce AGR2 degradation via autophagy. Moreover, etravirine diminished proliferation, migration, and invasion in vitro. Moreover, in an orthotopic xenograft mouse model, the combination of etravirine and paclitaxel significantly suppressed cancer progression and metastasis. This drug may be a promising therapeutic agent for the treatment of ovarian cancer.

## 1. Introduction

Cancer is the fourth-leading cause of death, and ovarian cancer is the second-leading cause of death in gynecologic cancers [1,2]. It is estimated that over 295,000 new diagnoses of ovarian cancer and over 184,799 associated deaths occur worldwide annually [3,4]. The standard treatment for ovarian cancer is exploratory surgery for primary staging or debulking, followed by six rounds of chemotherapy [5]. With this approach, the overall five-year survival rate for ovarian cancer is approximately 46%. The prognosis of ovarian cancer is strongly associated with the stage at diagnosis as per the International Federation of Gynecology and Obstetrics classification [6,7]. Therefore, a novel and effective anticancer drug is needed.

Human anterior gradient protein 2 homolog (AGR2), which is a protein belonging to the protein disulfide isomerase family, resides in the endoplasmic reticulum [8]. Beside, AGR2 is also found in plasma membrane, cytoplasm, and the extracellular environment [8,9]. AGR2 is overexpressed in numerous cancers, including breast [10,11], gastric [12], prostate [13,14], lung [15,16], and ovarian cancers [17,18]. Moreover, AGR2 is closely related to carcinogenesis [19], chemoresistance [20,21], and poor prognosis [22,23]. AGR2 interacts with vascular endothelial growth factor (VEGF) to facilitate angiogenesis [24]. Notably, AGR2 can directly bind to VEGF and fibroblast growth factor 2 (FGF2), thus leading to enhanced metastasis and tumor progression [25]. Several studies have identified AGR2 as a potential oncology biomarker [26] and a potential drug target in lung cancer [16], cervical carcinoma [27], and chronic myelogenous leukemia [28].

One study reported that the incidence of neoplastic lesions diminished in antiretroviral therapy (ART) recipients, such as Kaposi’s sarcoma patients [29,30], non-Hodgkin’s lymphoma patients [31,32], and women with cervical cancer who are living with human immunodeficiency virus (HIV) [33,34]. These results support the feasibility of antineoplastic treatment using ART.

Herein, we show that etravirine, a non-nucleoside reverse transcriptase inhibitor, could induce AGR2 degradation via autophagy when used in HIV therapy. Moreover, a combination treatment using etravirine and paclitaxel effectively suppressed ovarian tumor progression in an orthotopic xenograft mouse model.

## 2. Results

### 2.1. Etravirine Induces AGR2 Degradation via the Induction of Autophagy

**Figure 1:**
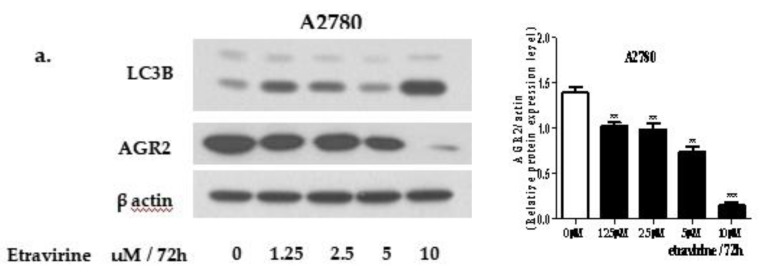

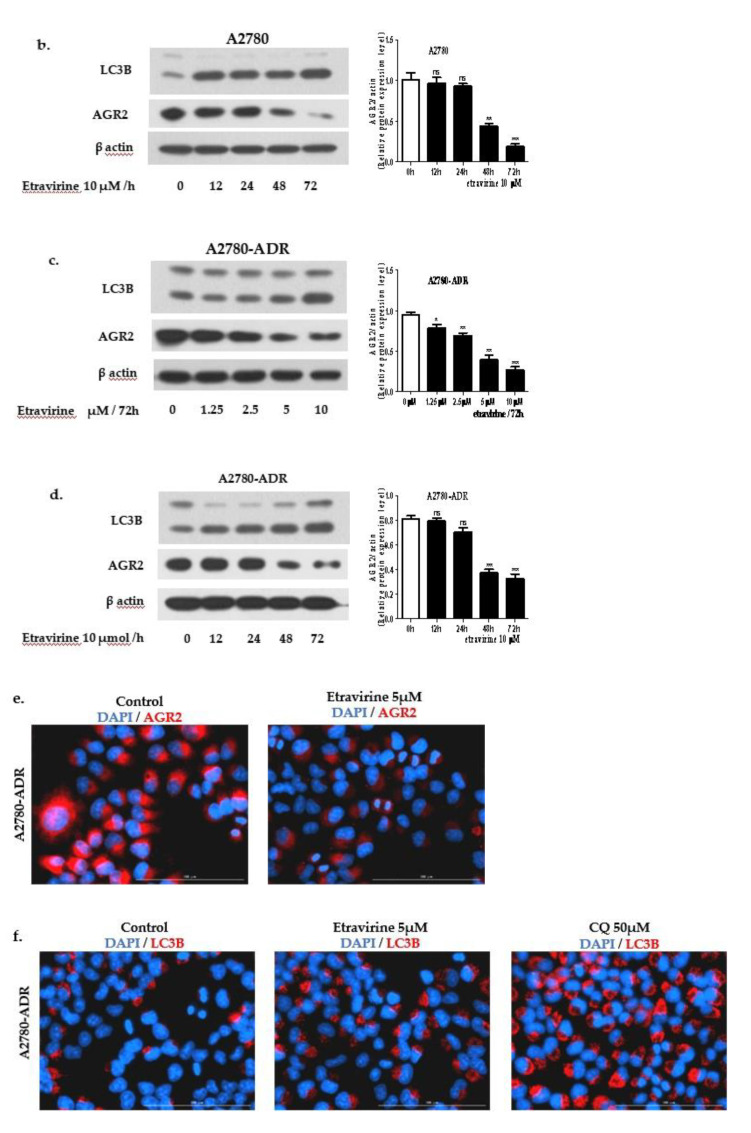

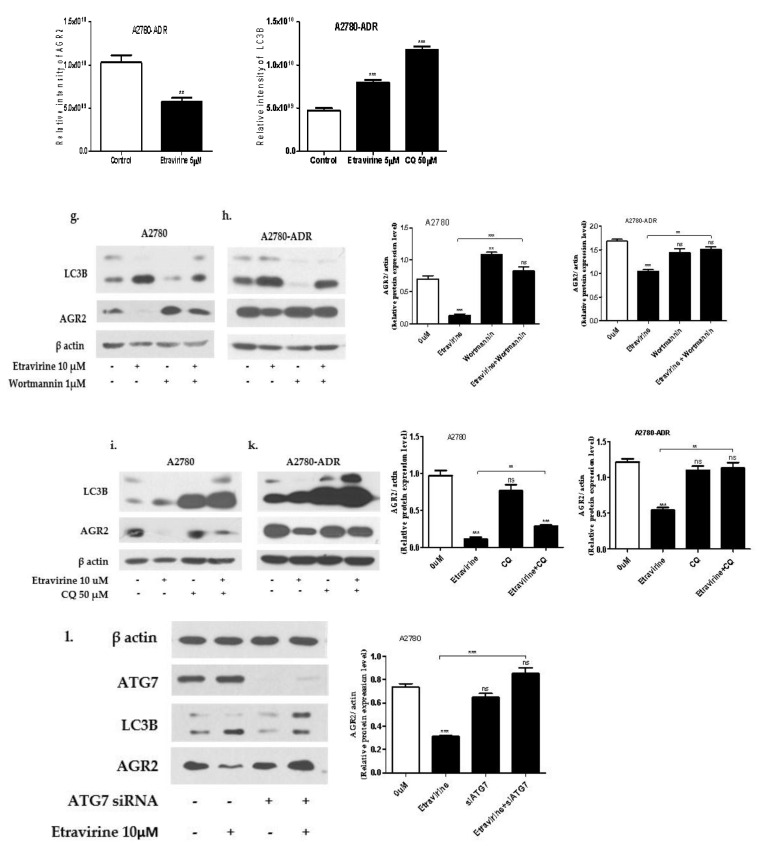
Etravirine induces AGR2 degradation by autophagy. (**a**–**d**) Western blot analysis showed the effect of etravirine on AGR2 expression and LC3B expression. (**e**,**f**) Immunofluorescence analysis showed the expressions of AGR2 and LC3B in A2780-ADR cells after treatment with 5 µM etravirine for 48 h and 50 µM CQ for 24 h by using Lion Heart FX automated microscope (40×). Scale bar 100 µm. (**g**,**h**) A2780 and A2780-ADR cells were exposed with wortmannin (PI3K inhibitor) 1 µM for 24 h prior to etravirine 10 µM for 72 h. (**i**,**k**) A2780 and A2780-ADR cells were pretreated with 50 µM CQ for 24 h and then treated with 10 µM etravirine for 72 h. (**l**) Knockdown of ATG7 by 100 nM siRNA for 72 h in A2780 cells and then treated with etravirine 10 uM for 72 h, AGR2 and LC3B expression were detected by Western blot. Data are shown as mean ± SEM. * p < 0.05, ** *p* < 0.01, *** *p* < 0.001.

AGR2 is overexpressed in several human cancers, including lung cancer, colon cancer, breast cancer, and ovarian cancer. In this study, we confirmed AGR2 expression in A2780 and A2780-ADR cells. Intracellular and extracellular AGR2 is highly expressed in A2780-ADR cells compared with A2780 cells, which were observed by Western blot assay and intracellular staining assay (Supplementary Figure S1). Western blot analysis showed that AGR2 levels significantly reduced after etravirine treatment in a dose- and time-dependent manner (Figure 1a–d). Additionally, the immunofluorescence analysis of AGR2 staining showed that etravirine decreased AGR2 expression in A2780-ADR cells (Figure 1e). To confirm whether AGR2 degradation is required for autophagy, we examined LC3B accumulation after etravirine treatment. As shown in Figure 1a–d, etravirine dramatically increased LC3B protein levels in a dose- and time-dependent manner in A2780 and A2780-ADR cells. Moreover, etravirine induced LC3B formation in A2780-ADR cells (Figure 1f). Using the Cyto-ID autophagy detection kit, an increase of autophagosome formation was observed in cells treated with etravirine (Supplementary Figure S2a). The ATG/ULK plays an essential role in the initiation of autophagy. Western blot analysis showed that ULK1 levels increased after etravirine treatment in A2780 and A2780-ADR cells (Supplementary Figure S2b).

**Figure 2:**
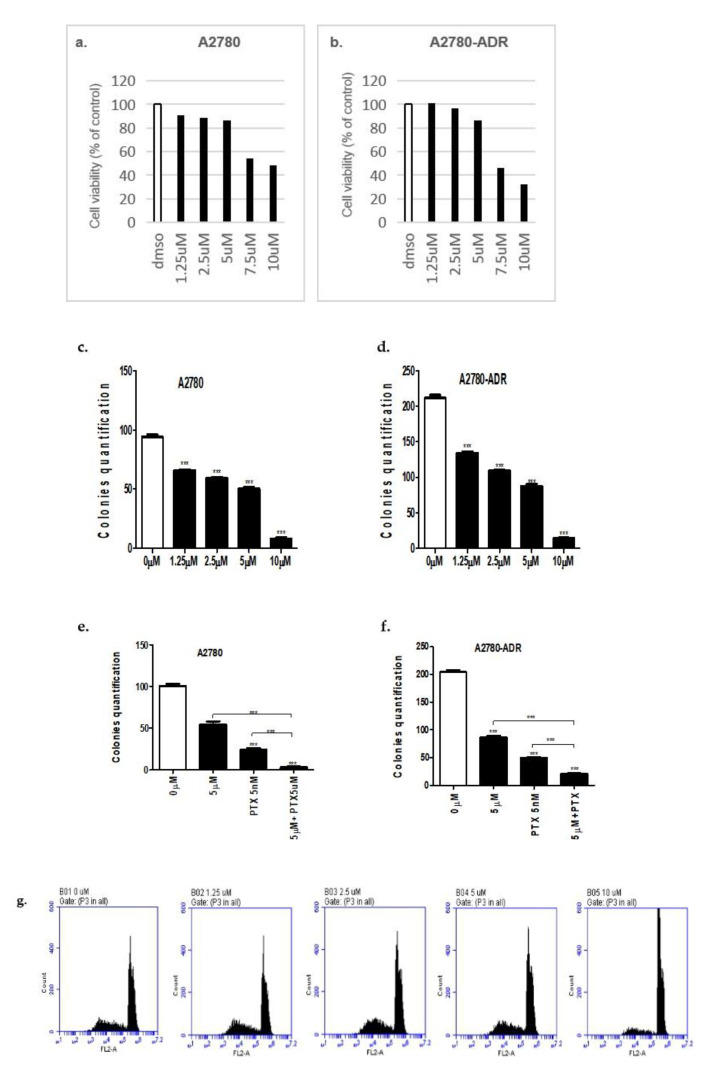

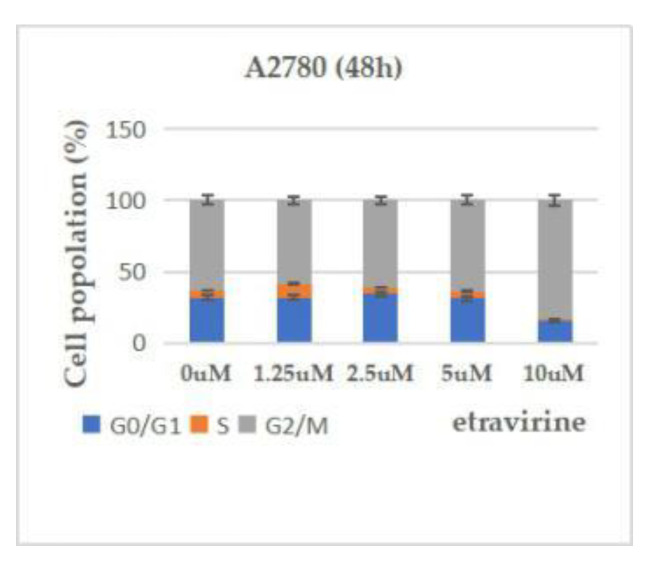
Effect of etravirine on ovarian cancer cell proliferation. (**a**,**b**) A2780 and A2780-ADR were treated with several concentrations of etravirine (up to 10 µM) for 48 h, and cell viability was determined by CCK8 assay (data are presented as percentages versus the control). (**c**,**d**) Etravirine inhibits the colony formation ability of A2780 and A2780-ADR cells. (**e**,**f**) Synergistic effect of 5 nM paclitaxel and 5 µM etravirine on colony growth in A2780 and A2780-ADR cells. (**g**) A2780 cells were treated with different concentrations of etravirne for 48 h, and cell cycle arrest were observed by using flow cytometry. All data are representative of the three independent experiments. Data are shown as mean ± SEM. *** *p* < 0.001.

To clarify the role of autophagy in AGR2 degradation, we co-treated etravirine with wortmannin, which is a phosphoinositide 3-kinase (PI3K) inhibitor. The inactivation of autophagy by wortmannin reduced LC3B expression and reversed AGR2 expression, which was suppressed by etravirine treatment (Figure 1g,h). Additionally, combining etravirine and CQ effectively restored AGR2 expression and augmented LC3B expression compared with etravirine treatment alone (Figure 1i,k). ATG7 plays a crucial role in autophagosome formation [35,36]. We attenuated the expression of ATG7 by using ATG7 siRNA. As expected, knockdown of ATG7 could interrupt the autophagosome formation and reverse etravirine-induced degradation of AGR2 protein (Figure 2). These data demonstrated that etravirine reduced AGR2 expression via autophagy-mediated lysosomal protein degradation.

### 2.2. Etravirine Reduces Cell Proliferation in Ovarian Cancer

To investigate the effect of etravirine on proliferation ability, cell viability assay and colony formation assay were performed. As shown in Figure 2a–d, Supplementary Figure S3 and Supplementary Figure S4a,b, etravirine impaired cell viability in A2780, A2780-ADR, SKOV3, OVCAR8 cells and colony formation in A2780 and A2780-ADR cells. Moreover, combining etravirine with paclitaxel dramatically inhibited the colony formation ability of A2780 and A2780-ADR cells compared with etravirine alone and paclitaxel alone (Figure 2e,f and Supplementary Figure S4c,d). Additionally, A2780 cells were treated with different concentrations of etravirine for 48 h. Cell cycle analysis showed that etravirine increased the percentage of G2/M phase cells compared to control cells (Figure 2g). These data proved that etravirine effectively inhibited ovarian cancer cell proliferation in vitro.

### 2.3. Etravirine Suppresses Spheroid Formation

**Figure 3:**
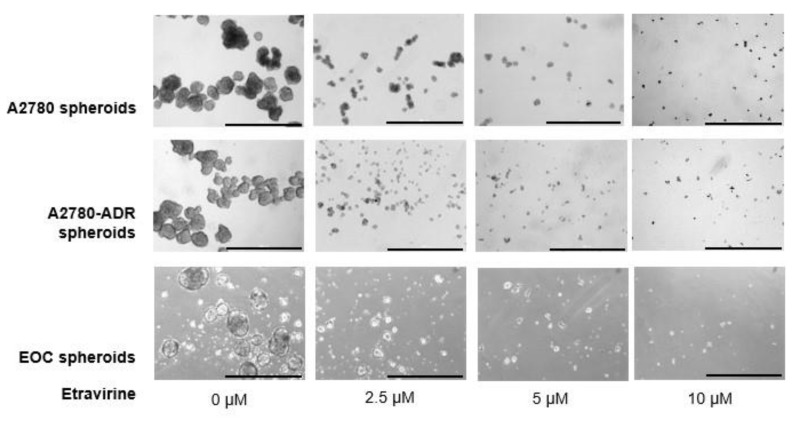

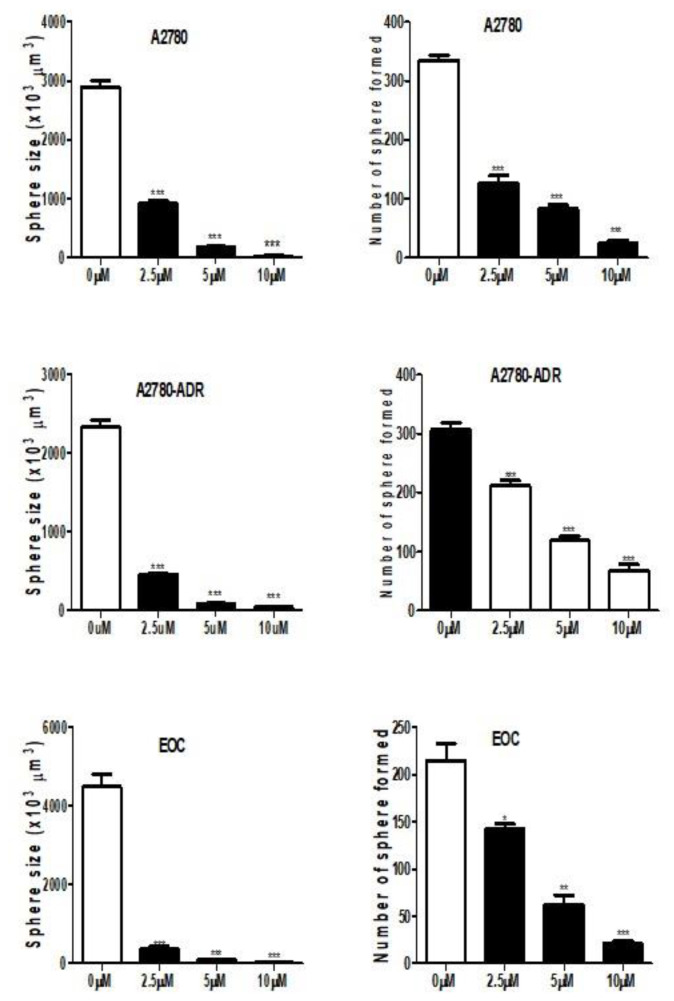
Etravirine inhibits ovarian cancer spheroid formation. Representative photomicrographs illustrate the effect of etravirine on spheroid growth at the indicated concentrations every two days for seven days in A2780, A2780-ADR, and EOC. Spheroid diameter and quantity were measured. Scar bar = 1000 µm. Data are shown as mean ± SEM. * p < 0.05, ** p < 0.01, ***p < 0.001.

To determine the effect of etravirine on ovarian cancer spheroid formation, A2780, A2780-ADR stem cell-like cells, and EOC spheroids were treated with etravirine at the indicated concentrations for seven days. As shown in Figure 3, etravirine significantly diminished spheroid formation.

### 2.4. Etravirine Inhibits HUVEC Tube Formation

**Figure 4:**
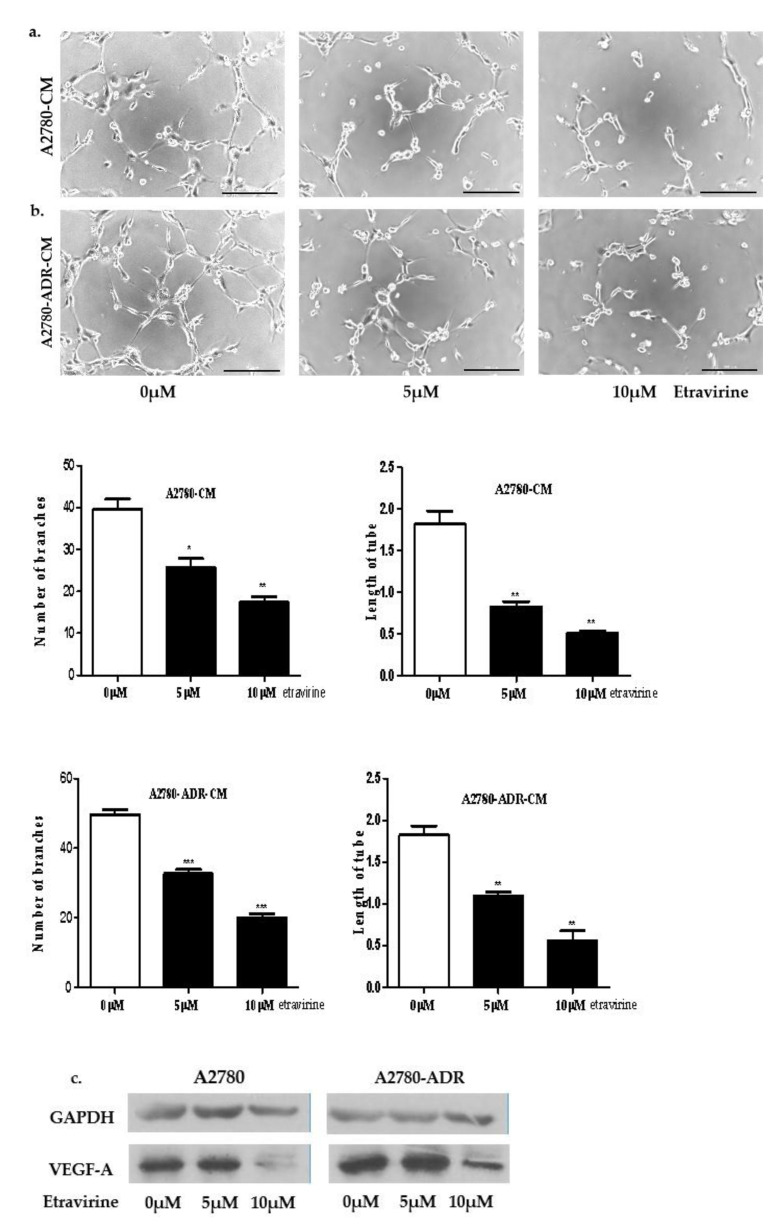
Etravirine inhibits HUVECs tube formation. (**a**,**b**) Representative images of tube formation. Etravirine with the indicated concentrations inhibit the tube formation abilities of A2780- and A2780-ADR-conditioned media. Quantitative analysis of the number of tubes and the total length of tubes. Scar bar = 200 µm. Data are shown as mean ± SEM.* p < 0.05, ** p < 0.01, *** *p* < 0.001. (**c**) Western blot analysis showing the expression of VEGF-A after treatment with etravirine (0, 5, and 10 µM) for 72 h.

To test whether etravirine can suppress angiogenesis in ovarian cancer, we observed HUVEC tube formation under media conditions with etravirine at the indicated concentrations. In A2780 and A2780-ADR cells, the number of tubes and the total length of the branch decreased after etravirine treatment (Figure 4a,b). Moreover, Western blot analysis indicated that VEGF-A expression was also reduced after etravirine treatment (Figure 4c). Our analysis suggested that etravirine remarkably inhibited HUVEC tube formation and decreased VEGF-A expression.

### 2.5. Etravirine Inhibits Ovarian Cell Migration and Invasion

**Figure 5:**
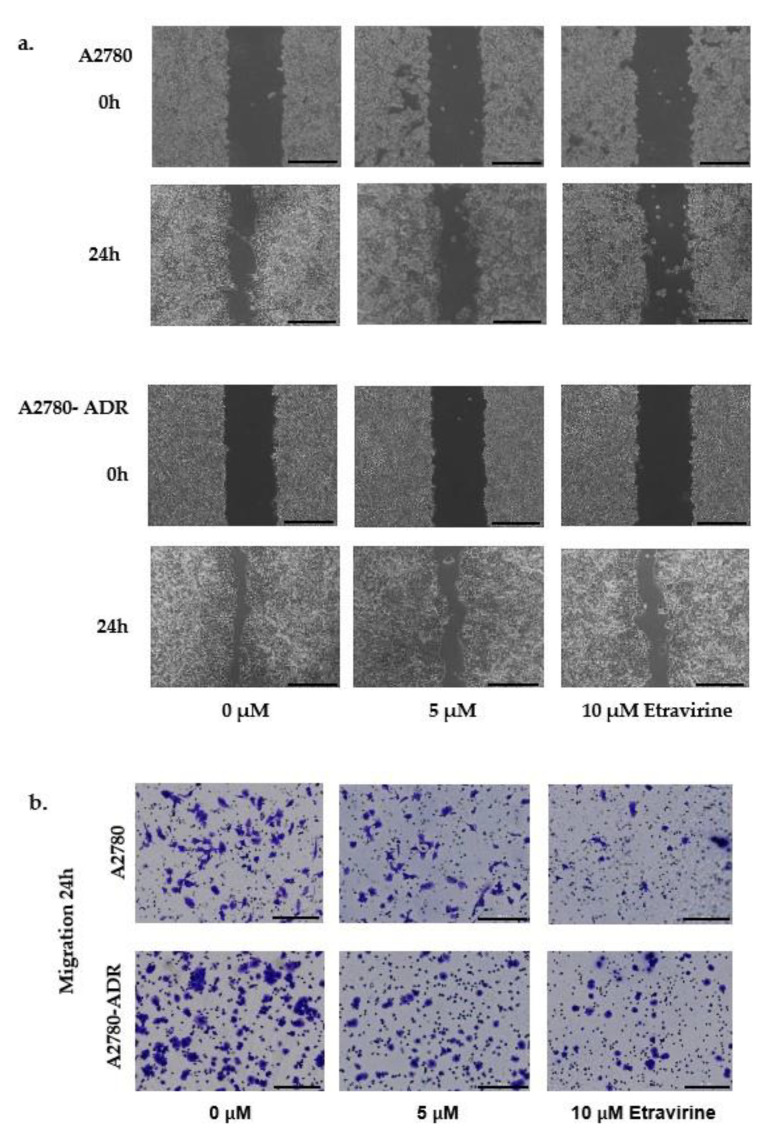

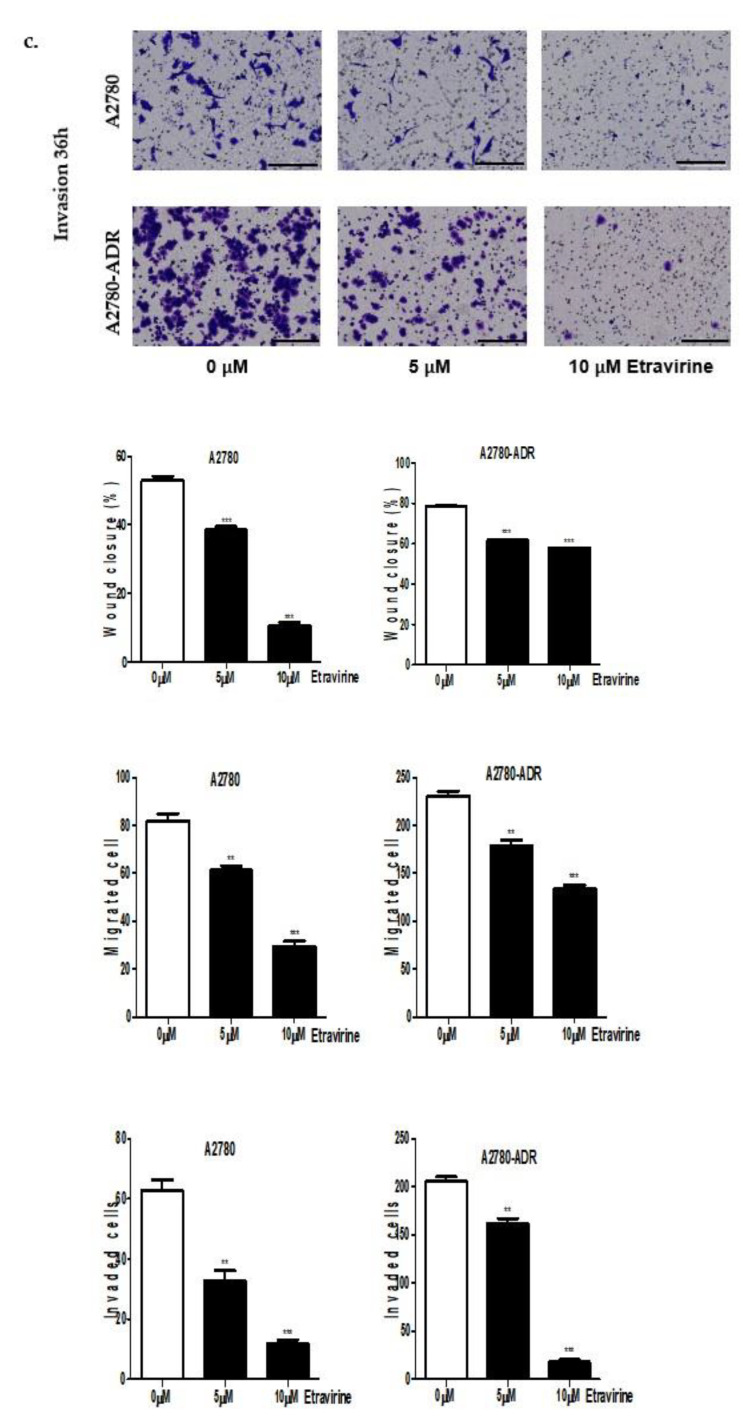
Etravirine inhibits ovarian cell migration and invasion. (**a**) The wound-healing assay showed that the wound closure rate significantly decreased with 5 and 10 µM etravirine compared to the untreated control in the 24 h time course in A2780 and A2780-ADR cells. (**b**) Transwell migration assay demonstrated that etravirine reduced the number of migrated cells in the group treated with 5 and 10 µM etravirine compared with that in the control group. (**c**) Transwell invasion assay illustrated that etravirine inhibited the number of invaded cells compared to the control. Scar bar = 200 µm. Data are shown as mean ± SEM.* *p* < 0.05, ** *p* < 0.01, *** *p* < 0.001.

Cancer metastasis is the central cause of cancer therapy failure and mortality. Numerous reports have indicated that AGR2 promotes cell migration and invasion, and we found that etravirine could suppress AGR2 expression in ovarian cancer. Thus, we verified the effects of etravirine on migration and invasion in ovarian cancer cells by using a transwell assay and wound-healing experiments. First, a wound-healing scratch was observed at 0 and 24 h. Etravirine remarkably delayed the wound closure percentage in the presence of etravirine compared to that in the control (Figure 5a). Thereafter, 5 × 10^4^ A2780 and A2780-ADR cells suspended in 100 µL high-glucose, serum-free DMEM were seeded into the upper transwell chamber with an 8-µm pore size (Corning), whereas the bottom transwell chamber was filled with 600 µL DMEM containing 10% FBS with etravirine at the indicated concentrations for 24 h. Etravirine significantly inhibited the migration of both A2780 and A2780-ADR cells (Figure 5b). Cell invasion was examined using Matrigel-coated transwell plates. Moreover, etravirine also inhibited the invasion of A2780 and A2780-ADR cells (Figure 5c). These data revealed that etravirine strongly inhibited ovarian cell migration and invasion.

### 2.6. Etravirine Suppresses Tumor Progression in an Orthotopic Ovarian Cancer Mouse Model

**Figure 6:**
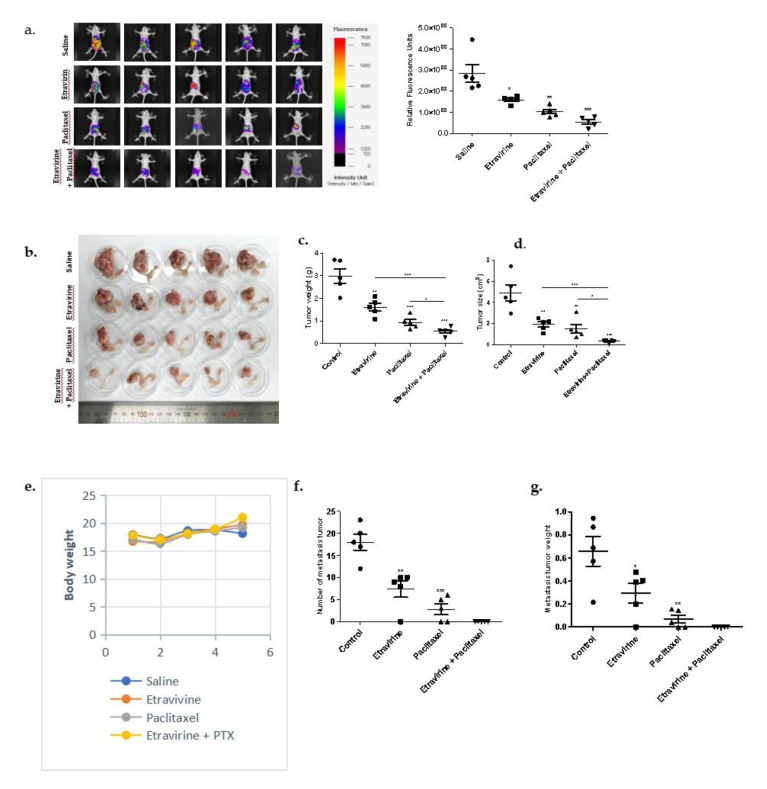

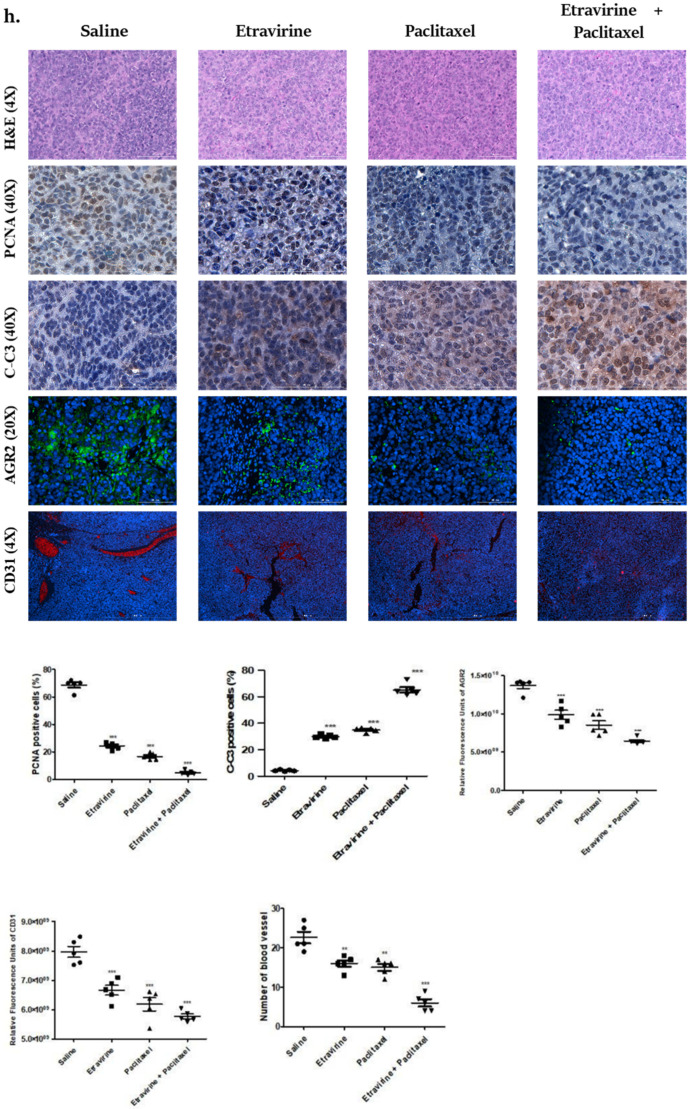
Combination treatment of etravirine and paclitaxel suppressed tumor growth and metastasis in the A2780 orthotopic xenograft model. (**a**,**b**) A2780 cells were injected into the ovarian bursa sac of nude mice (five mice per group). Randomized mice were treated with control (saline), etravirine (100 mg/kg × three times per week by i.p. injections), paclitaxel (15 mg/kg once a week), and a combination group for three weeks. Representative fluorescence images of female nude mice after three weeks of treatment. (**c**,**d**) Tumor weight and tumor size were measured at the end of the treatment. (**e**) Mice’s body weights were monitored weekly. (**f**,**g**) Metastatic tumors were counted and measured. (**h**) Representative H&E staining, IHC images, and quantification of cell proliferation marker PCNA, AGR2, angiogenesis marker CD31, and apoptosis marker cleaved caspase-3. Data are presented as mean ± SEM. One-way ANOVA and t-test analyses. * *p* < 0.05, ** *p* < 0.01, *** *p* < 0.001.

To observe the inhibitory effect of etravirine on the primary tumor in ovaries and tumor metastasis in mice, we used an orthotopic ovarian cancer mouse model and injected A2780 cells into the ovarian bursa sac of six-week-old female Balb/c-nude mice. Thereafter, the mice were treated with etravirine 100 mg/kg three times a week, paclitaxel 15 mg/kg once a week, and a combination of the two drugs for three weeks. At the end of the treatment period, tumor growth and tumor metastasis were observed using live mouse imaging with NpFlamma HGC ICG. Representative images and quantification of the fluorescent signal showed the significant inhibition of tumor growth by etravirine and the combined treatment of etravirine and paclitaxel (Figure 6a). Figure 6b–d indicates the remarkable suppression of the primary tumor in the ovaries by etravirine treatment and the combination treatment, as evidenced by the changes in tumor size and weight. The results in Figure 6e reveal that the body weight of the treated mice remained almost unchanged. Furthermore, the results indicated a significant decrease in the number of tumors and in the weight of tumor metastasis in mice treated with etravirine, paclitaxel, and the combination treatment compared with the control group (Figure 6f,g). The IHC assay revealed that the combination treatment mostly eliminated the positive PCNA-stained cells compared to the control group and etravirine group (Figure 6h). Moreover, AGR2 and CD31-positive staining was also decreased by etravirine treatment and the combination treatment. Cleaved caspase-3-positive staining was significantly increased in the treatment group compared with the control group (Figure 6h). Furthermore, no histological abnormalities were observed in major organs such as the liver, heart, lung, kidney, and spleen such as: hemorrhage, hematoma formation, necrosis, and infiltration of inflammatory cells (Supplementary Figure S5). These data indicated that the inhibitory effect of etravirine could impede ovarian cancer growth and cancer metastasis without toxic side effects to the vital organs.

## 3. Discussion

Metastasis ovarian cancer is an advanced stage malignancy in which ovarian cancer cells spread from the primary tumor to distant areas such as the peritoneal cavity, omentum [37,38], liver [39], intestines, spleen [40], lungs [41,42], brain [43,44], and lymph nodes outside the abdomen [45]. Cancer cells must migrate and invade through the extracellular matrix into the blood vessels and lymphatic system, and then extravasate from the vessel to form distant sites [46].

A study indicated that etravirine, which was approved by the FDA because of its efficacy and safety in HIV patients [47], induced the differentiation of SKOV3 ovarian cancer cells [48]. In the current study, etravirine significantly inhibited cell proliferation, migration, and invasion in A2780 and A2780-ADR cell lines. Furthermore, etravirine was synergistic with paclitaxel in inhibiting colony formation and tumor growth in an orthotopic mouse model. Surgery is often the initial treatment in ovarian cancer, followed by chemotherapy for six cycles. However, chemotherapy is often limited by toxic side effects to the vital organs. We also observed that treatment with etravirine did not exert toxic effects on the major organs, such as the heart, lungs, kidneys, liver, and spleen. These findings indicate that etravirine has interaction potential with chemotherapy in ovarian cancer.

AGR2 is an endoplasmic protein that is secreted into the extracellular space. Accumulating evidence has shown that AGR2 plays a crucial role in tumor growth [49], cell proliferation, and cell migration [50]. AGR2 is elevated in patients with metastatic prostate cancer [51], pituitary adenomas [52]. AGR2 could serve as a biomarker for the prognosis of metastasis and chemotherapeutic response in breast cancer [53,54], lung cancer [15], gastric cancer [12], esophageal squamous cell carcinoma [55]. Moreover, the present study demonstrated that targeting AGR2 is a potential anticancer approach [16,27]. Secreted AGR2 can interact with VEGF-A via the formation of disulfide bonds [24]. Extracellular AGR2 induces angiogenesis by binding to VEGF-A and FGF2, thus leading to migration and metastasis in cancer [25]. In the present study, we revealed that etravirine successfully suppressed HUVEC tube formation in A2780 and A2780-ADR cell lines. Additionally, etravirine decreased the AGR2 levels in ovarian cancer cells in a dose- and time-dependent manner.

Autophagy is a major intracellular degradation program that initiates autophagosome formation; autophagosomes then fuse with lysosomes to form autolysosomes and allow degradation [56]. During autophagosome formation, the LC3-A conjugated with phosphatidylethanolamine by ATG7, ATG3, and ATG12–ATG5–ATG16 complex is converted to LC3-B, which is associated with the phagophore. LC3B is essential for autophagosome information and maturation [57]. A recent report revealed that proteasome inhibitor MG132/bortezomib suppressed AGR2 expression in an ER stress-independent manner and proteasome inhibitor-induced AGR2 degradation via activation of autophagy in lung cancer [58]. Interestingly, our study demonstrated that etravirine, a non-nucleoside reverse transcriptase inhibitor, increased LC3B levels, LC3B fluorescence and Cyto-ID fluorescence, thus indicating increased autophagosome formation. Wortmannin, which is a PI3K inhibitor and AGT7 knockdown blocks autophagosome formation. Furthermore, CQ inhibits autophagy by blocking the fusion of autophagosomes with lysosomes [59]. A combination treatment involving etravirine and an autophagy inhibitor can reverse AGR2 expression. These findings indicate that autophagy is essential for AGR2 expression in ovarian cancer.

In summary, etravirine effectively suppresses ovarian cancer progression by promoting AGR2 autophagy degradation. These results indicate that etravirine may be used in neoadjuvant chemotherapy in ovarian cancer.

## 4. Materials and Methods

### 4.1. Cell lines and Culture Conditions

The human ovarian cancer cell lines A2780, A2780-ADR (Adriamycin resistant), SKOV3, and OVCAR8 were kindly supplied by Jae Ho Kim from the Pusan National University, South Korea. They were cultured in high-glucose Dulbecco’s modified Eagle medium (DMEM) (Welgene, South Korea) supplemented with 10% fetal bovine serum (FBS) (Gibco, Thermo Fisher Scientific, Hillsboro, OR, USA) and 100 µg/mL penicillin–streptomycin (Welgene, South Korea) at 37 °C and 5% CO_2_ in a humidified incubator. The cells were passaged every three days by using trypsin–EDTA (Welgene, South Korea). A2780-ADR cells were isolated from the human ovarian cancer cell line A2780. A2780 cells were exposed to adriamycin in a stepwise manner. The adriamycin concentration was 1 nM, and the surviving cells were treated with two concentrations. The process repeated up to 128 nM adriamycin to increase the resistance to adriamycin [60]. Primary human umbilical vein endothelial cells (HUVECs) were purchased from ScienCell Research Laboratories (CA, USA). HUVECs were cultured in an EGM-2 bullet kit system (Lonza, Walkersville, MD, USA), which contains endothelial basal medium 2, 5% FBS, human VEGF, human basic fibroblast growth factor, human epidermal growth factor (EGF), human insulin-like growth factor-1, ascorbic acid, GA-1000, and 100 µg/mL penicillin–streptomycin (Welgene, South Korea). These cells were cultured in a humidified atmosphere of 5% CO_2_ at 37 °C.

### 4.2. Drugs and Reagents

Etravirine (T2551), paclitaxel (T1912), and wortmannin (T6283) were purchased from TargetMol (Boston, MA, USA). Chloroquine (CQ, C6628), Immobilon Crescendo Western HRP substrate (WBLUR0500), Propidium iodide (P4170), Ribonuclease A (RNase A) (R6513), siRNA universal negative control (SIC001) were purchased from Sigma-Aldrich (St. Louis, MO, USA). Antibodies against AGR2 (ab76473), LC3B (ab48394), and CD31 (ab76533), anti-ATG7 (ab53255) were purchased from Abcam (Life Technologies Corporation, Carlsbad, CA, USA). Antibodies against GAPDH (sc-32233), VEGF (A-20, sc-152), and beta actin (sc-47778) were purchased from Santa Cruz Biotechnology (Dallas, TX, USA). Antibodies against cleaved caspase-3 (Asp175, 9664), ATG7 siRNA (6604S), ULK1 (8054S), and PCNA (D3H8P, 13110) antibodies were purchased from Cell Signaling Technology (Boston, MA, USA). NpFlamma HGC ICG (PNC1501) was purchased from BioActs (Incheon, South Korea).

### 4.3. Quantitative Real-Time PCR (RT-PCR)

Total RNA was isolated using TRIzol reagent (Thermo Fisher Scientific) according to the manufacturer’s protocol. The RNA concentration was determined using a NanoDrop 200C. cDNA synthesis was performed using a PrimeScript 1st Strand cDNA Synthesis Kit (Takara, Shiga, Japan). Specific primers for AGR2 (forward primer, 5′-ATCCAGAAATTGGCAGAGGACTTTGTC-3′, reverse primer, 3′-CTAACTGTCAGAGATGGGTCAACAAACATAATC-5′) and GAPDH (forward primer, 5′-TGCACCACCAACTGCTTAGC-3′, reverse primer, 3′-GGCATGGACTGTGGTCATGAG-5′) were used. Quantitative RT-PCR was performed with SYBR green (Roche, Basel, Switzerland) according to the manufacturer’s instructions on a LightCycler 96 RT-PCR system (Roche).

### 4.4. Western Blot Analysis

Treated and untreated cells were harvested after washing twice with ice-cold PBS. Cells were then lysed in Pro-prep (17081, iNtRON Biotechnology). Total cellular protein concentrations were quantified using a BCA assay kit (Thermo Fisher Scientific). Load samples containing equal amounts of protein in the SDS-PAGE wells were electrophoresed as previously reported [61]. The membranes were incubated with primary antibodies against AGR2 (ab76473), LC3B (ab48394), VEGF (A-20, sc-152), beta actin (sc-47778), and GAPDH (sc-32233) overnight at 4 °C, followed by incubation with secondary antibodies, goat anti-rabbit IgG-horseradish peroxidase (HRP 1:5000; ADI-SAB-100; Enzo Life Sciences, Farmingdale, NY, USA), or goat anti-mouse IgG-HRP (1:5000; ADI-SAB-100; Enzo Life Sciences) for 1 h at room temperature. Protein signals were developed using an Immobilon Crescendo Western HRP substrate (WBLUR0500) and were exposed on auto-radiography films.

### 4.5. Quantification of AGR2 Secretion

Conditioned media were collected and mixed with acetone at a 1:4 ratio and then stored at −80 ℃ for 1 h. The mixture was centrifuged at 12,000 rpm for 30 min, and the supernatant was removed and eluted with Pierce IP buffer (Thermo Fisher Scientific). After quantifying the protein, the sample was used for Western blot analysis, as mentioned above.

### 4.6. Intracellular Staining

Cells were fixed with 4% formaldehyde for 5 min and permeabilized with 0.1% PBS-Tween for 20 min. The cells were then incubated in 1x PBS/10% normal goat serum/0.3 M glycine to block non-specific protein–protein interactions followed by incubation with the antibody AGR2 Alexa Flour 488 1/500 (ab199044, Abcam, Life Technologies Corporation, Carlsbad, CA, USA) for 30 min. The flow cytometer used for this analysis was BD Bioscience Accuri C6.

### 4.7. Immunofluorescence Assay

A2780-ADR cells (5 × 10^4^ cells/well) were seeded on poly-L-lysine-coated 24-well slides and were incubated overnight at 37 °C and 5% CO_2_. These cells were treated with 5 µM etravirine for 48 h and 50 µM CQ for 24 h. Thereafter, these cells were fixed with 100% methanol (chilled at −20 °C) at room temperature and washed three times with ice-cold PBS. These cells were permeabilized with PBS containing 0.25% Triton X-100 for 10 min and blocked with 1% bovine serum albumin (BSA) (Roche, Germany), 22.52 mg/mL glycine, and 0.1% Tween 20 in PBS at room temperature. These cells were stained with primary antibodies LC3B and AGR2 in 1% BSA and 0.1% Tween 20 in PBS overnight at 4° C in the dark. After washing three times with ice-cold PBS, the cells were stained with anti-rabbit Alexa Fluor 594 dye (Life Technologies, Carlsbad, CA, USA) for 1 h at room temperature in the dark. Slides were mounted using ProLong Diamond Antifade Mountant with DAPI (Thermo Fisher Scientific, Hillsboro, OR, USA). Images were captured using a 40× objective lens on a Lion Heart FX automated microscope (Biotek, Winooski, VT, USA) (scale bar = 100 µm).

### 4.8. Cell Viability and Colony Formation Assay

The cytotoxicity of etravirine on A2780 and A2780-ADR cells was assessed using a CCK assay kit (Dongin LS, Seoul, South Korea). First, ovarian cancer cells were plated in 96-well plates at 2500 cells per well and grown overnight. The cells were then replaced with a fresh medium containing various final concentrations of etravirine (0–10 µM) for 72 h. Thereafter, the absorbance was measured using a SUNRISE-microplate reader at a wavelength of 450 nm.

For colony formation, A2780 and A2780-ADR cells were seeded into 6-well plates (1000 cells per well) and were incubated with various concentrations of etravirine (0–10 µM), paclitaxel (5 nM), and a combination of 10 µM etravirine and 5 nM paclitaxel. After seeding for 14 days, the colonies were fixed with 4% paraformaldehyde for 10 min and stained with 0.05% crystal violet in 25% methanol for 30 min at room temperature. The solution was washed several times with distilled water to remove excess dye. The colonies were imaged and counted using ImageJ software. Experiments were performed in triplicate for each group.

### 4.9. Cell Cycle Assay

A2780 cells were seeded into 6-well plates (5 × 10^5^ per well) and were incubated overnight at 37 °C, 5% CO_2_. These cells were treated with several concentrations of etravirine (0-10uM) for 48 h. These cells were collected, washed with PBS, and 1x10^6^ cells were fixed with cold 70% ethanol for 1 h on ice. Each sample was washed twice with cold PBS, and then added Rnase A and propidium iodide, mixed and incubated for 1 h, at 4 °C in the dark. Cell cycle distributions were analyzed by flow cytometry (BD Bioscience Accuri C6).

### 4.10. RNA Interference Assay

A2780 cells were seeded into the 6-well plates (3 × 10^5^ per well) overnight in the incubator. These cells were transfected with 100 nM siATG7 for 72 h, or negative control by using Lipofectamine RNAiMax Reagent (Invitrogen, USA). After that, these cells were treated with etravirine 10 uM for 72 h. ATG7 were confirmed by Western blot with the specific antibody.

### 4.11. Spheroid Culture Conditions

Epithelial ovarian cancer cells (EOCs) were gifted by Jae Ho Kim (Pusan National University, South Korea). Isolation of sphere-forming cancer stem cells population from tumor tissue of ovarian cancer patients is described in previous reports [62]. Spheroid cultures of the ovarian cancer cell lines were cultured in neurobasal medium (Gibco) containing 1X B27 (Gibco), 10 ng/m basic fibroblast growth factor (Peprotech, Rocky Hill, NJ, USA), 20 ng/mL EGF (Peprotech), HEPES (Gibco), Glutamax (Gibco), and penicillin/streptomycin (Welgen). Ultra-low attachment plates (Corning, Inc., Corning, NY, USA) were used to culture and propagate cancer stem cells.

### 4.12. Effect of Etravirine on Spheroid Growth

A2780, A2780-ADR, and EOCs were seeded into ultra-low attachment 6-well plates, with 5000 cells per well. Etravirine was administered at the indicated concentrations every two days for seven days. On days 5 and 7, the spheroids were captured using a Lion Heart FX automated microscope (Biotek, Winooski, VT, USA) and measured using ImageJ software. Sphere size was calculated using the following formula: sphere volume = 4/3πr^3^.

### 4.13. Transwell Assays

Cell migration assays were performed using a 24-well 8.0 µm polycarbonate transwell chamber (Corning, Inc., Corning, NY, USA), and Matrigel-precoated transwell inserts (BD Biosciences, San Jose, CA, USA) were used for the invasion assay.

For the migration assay, A2780 and A2780-ADR cells (5 × 10^5^ cells/mL) were suspended in high-glucose, serum-free DMEM; the upper chamber was filled with 100 µL cells, whereas the bottom chamber was filled with 600 µL DMEM containing 10% FBS with etravirine (0, 5, and 10 µM), followed by incubation at 37 °C and 5% CO_2_ for 24 h. After the incubation period, the insert was carefully removed. Cells on the lower side of the insert membrane were fixed with 4% paraformaldehyde for 10 min, followed by staining with 0.5% crystal violet in 25% methanol for 30 min at room temperature. The insert was washed twice with PBS for several seconds to remove excess dye. Cells in the upper part of the insert were removed by gently swiping with a cotton swab. Finally, the migrating cells were counted using a Lion Heart FX automated microscope (Biotek, Winooski, VT, USA). Experiments were performed in triplicate for each group.

For the invasion assay, all steps were the same as those described above, but the incubation time was 36 h.

### 4.14. Wound-Healing Assays

For the scratch wound-healing assay, A2780 and A2780-ADR cells (5 × 10^5^ cells/well) were seeded into six-well plates and scratched with a 1 mm scratch tip (SPL Life Sciences, Pocheon, South Korea) when the cells reached 90% confluence. Thereafter, the cells were lightly washed with PBS twice, and the scratched cells were cultured with etravirine (0, 5, and 10 µM). Wound closure was observed at 0 and 24 h under a microscope and was analyzed using ImageJ software.

### 4.15. Tube Formation Assays

A2780 and A2780-ADR cells were seeded into 6-well plates (5 × 10^5^ cells per well). When the cells reached 80% confluence, the cells were washed three times with PBS and then replaced with high-glucose, serum-free DMEM with etravirine at various final concentrations (0, 5, and 10 µM) for 48 h. Thereafter, 6 × 10^3^ primary HUVECs in 100 µL of conditioned medium derived from A2780 and A2780-ADR cells were seeded into 96-well plates that were coated with growth factor–reduced Matrigel (BD Biosciences, San Jose, CA, USA) (60 µL per well) and polymerized for 30 min at 37 °C. At 4 h after seeding, images were collected using a Lion Heart FX automated microscope (Biotek, Winooski, VT, USA). Tube length and branch formation were measured and analyzed using ImageJ software.

### 4.16. Animal Studies

Animal study protocol were carried out in accordance with the Pusan National University Institutional Animal Care and Use Committee (PNU-2019-2435).

For the orthotopic xenograft model, 1 × 10^6^ A2780 cells were injected into the ovarian bursa sac of six-week-old female Balb/c-nude mice. One week after injection, the mice were randomized into four groups (n = 5 mice per group), including the control group (saline 0.9%), etravirine group (100 mg/kg three times a week by i.p. injections), paclitaxel group (15 mg per kg once a week by i.p. injections), and combination group for three weeks. Body weights were measured weekly. At the end of the experiments, in vivo fluorescence imaging was performed using a fluorescence-labeled organism bio-imaging instrument (FOBI) (NeoScience Co., Ltd., Gyeonggi-do, South Korea). The mice were injected with 100 µL NpFlamma HGC ICG by i.p. injection (PNC1501, BioActs, Incheon, South Korea). Images and measurements of fluorescence signals were acquired and analyzed using NEOimage FOBI software. The primary tumor and major organs were dissected. The tumor size and weight were measured. The tumor volume (cm^3^) was calculated using the formula 0.5 × L × W^2^ (L = length, W = width).

### 4.17. Immunohistochemical (IHC) Staining and Hematoxylin and Eosin (H&E) Staining

Tissue samples isolated from an orthotopic xenograft model were used for histological analysis. Tissue samples were fixed with 4% paraformaldehyde, embedded in paraffin, and cut into 5-µm sections. Paraffin-embedded tissue was deparaffinized, rehydrated, and subjected to antigen retrieval. The slides were blocked and incubated with primary antibodies against AGR2 (1: 500, ab76473), CD31 (1: 200, ab76533), cleaved caspase-3 (1: 500, Asp175, 9664), and PCNA (1: 5000, D3H8P, 13110) overnight at 4 °C, followed by incubation with secondary antibodies, namely, goat anti-rabbit IgG-horseradish peroxidase (HRP 1:500; ADI-SAB-100; Enzo Life Sciences, Farmingdale, NY, USA) or goat anti-mouse IgG-HRP (1:500; ADI-SAB-100; Enzo Life Sciences), Alexa Flour 488 goat anti-rabbit IgG (A11008, Invitrogen), and Alexa Flour 647 goat anti-rabbit IgG (A32733, Invitrogen), for 1 h at room temperature. The primary tumor and major organ sections were stained with H&E. Images were visualized using a Lion Heart FX automated microscope (Biotek, Winooski, VT, USA).

### 4.18. Cyto-Id Autophagy Detection Assay

A2780-ADR cells were seeded into six-well plates (2 × 10^5^ cells/well) and were incubated overnight at 37 ℃ and 5% CO_2_. These cells were treated with etravirine (0 µM, 5 µM) for 72 h and 50 µM CQ for 24 h. Thereafter, these cells were stained with Cyto-id green detection reagent 2, Hoechst 33,342 nuclear stain (ENZ-KIT175, NY, USA), and incubated for 30 min at 37 ℃, protected from light. After washing three times with 1X assay buffer, these cells were analyzed by using Lion Heart FX automatic microscope (Biotek, Winooski, VT, USA) (scale bar = 100 µM).

### 4.19. Statistical Analysis

Statistical analysis was conducted by two-tailed unpaired Student’s t-test and one-way analysis of variance (ANOVA) using GraphPad Prism software version 7 (GraphPad, Inc., La Jolla, CA, USA). Data are presented as mean ± SEM. Statistical significance was set at *p* < 0.05.

## Supplementary Materials

The following are available online at https://www.mdpi.com/article/10.3390/ijms23020944/s1.

## Abbreviations

AGR2: anterior gradient protein 2 homolog; VEGF, Vascular endothelial growth factor; FGF2, Fibroblast growth factor 2; CQ, Chloroquine, CM, conditioned media.
