# Correction: Ly et al. Inhibitory Effect of Etravirine, a Non-Nucleoside Reverse Transcriptase Inhibitor, via Anterior Gradient Protein 2 Homolog Degradation Against Ovarian Cancer Metastasis. *Int. J. Mol. Sci.* 2022, *23*, 944

**DOI:** 10.3390/ijms27073216

**Published:** 2026-04-02

**Authors:** Thanh Truong Giang Ly, Jisoo Yun, Jong-Seong Ha, Yeon-Ju Kim, Woong-Bi Jang, Thi Hong Van Le, Vinoth Kumar Rethineswaran, Jaewoo Choi, Jae-Ho Kim, Sang-Hyun Min, Dong-Hyung Lee, Ju-Seok Yang, Joo-Seop Chung, Sang-Mo Kwon

**Affiliations:** 1 Laboratory for Vascular Medicine and Stem Cell Biology, Department of Physiology, Medical Research Institute, School of Medicine, Pusan National University, Yangsan 50612, Republic of Korea; lythanhtruonggiang@gmail.com (T.T.G.L.); jsyun14@hanmail.net (J.Y.); jongseong@pusan.ac.kr (J.-S.H.); twou1234@nate.com (Y.-J.K.); jangwoongbi@naver.com (W.-B.J.); lethihongvan25121978@gmail.com (T.H.V.L.); vinrebha@gmail.com (V.K.R.); wozh1304@naver.com (J.C.); 2 Convergence Stem Cell Research Center, Pusan National University, Yangsan 50612, Republic of Korea; jhkimst@pusan.ac.kr; 3 New Drug Development Center, Deagu Gyeongbuk Medical Innovation Foundation, Deagu 41061, Republic of Korea; shmin03@dgmif.re.kr; 4 Department of Obstetrics and Gynecology, Pusan National University Yangsan Hospital, Yangsan 50612, Republic of Korea; ldh0707@hanmail.net (D.-H.L.); yangandshin@gmail.com (J.-S.Y.); 5 Department of Hematology-Oncology, Pusan National University Hospital Medical Research Institute, Busan 49241, Republic of Korea

There was an error in the representative H&E staining images in Figure 6h in the original publication [1]. During the figure preparation process, an identical image was inadvertently duplicated across different experimental groups while arranging the representative image panels. The corrected Figure 6h appears below and has been reconstructed using the appropriate representative images obtained from the same experimental batch. The correct legend appears below. The authors apologize for any inconvenience caused and state that the scientific conclusions are unaffected. This correction was approved by the Academic Editor. The original publication has also been updated.

## Figures and Tables

**Figure 6:**
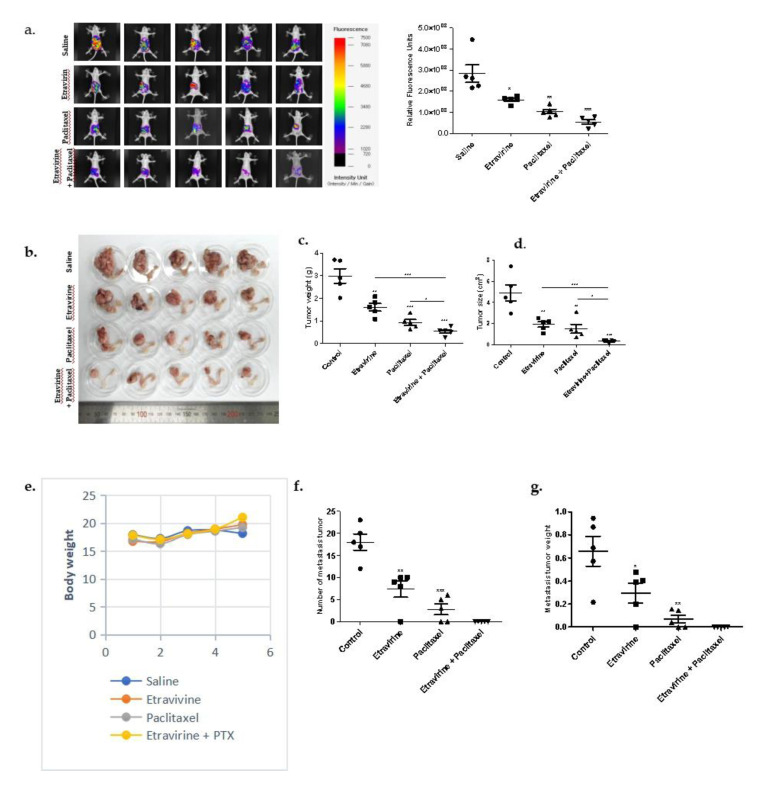

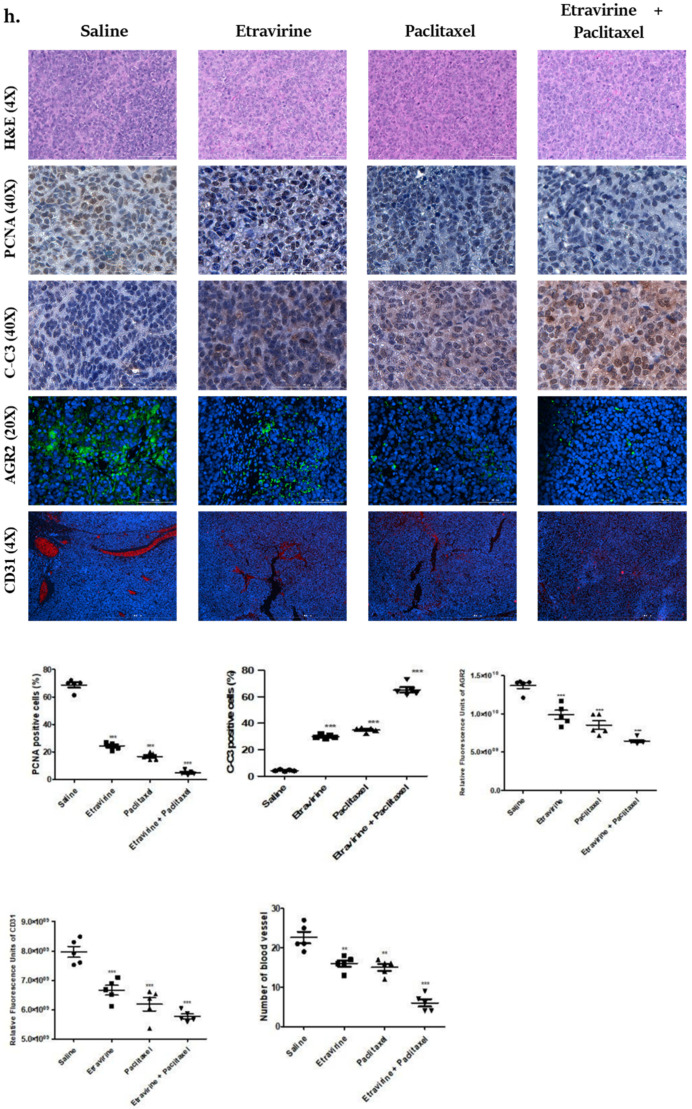
Combination treatment of etravirine and paclitaxel suppressed tumor growth and metastasis in the A2780 orthotopic xenograft model. (**a**,**b**) A2780 cells were injected into the ovarian bursa sac of nude mice (five mice per group). Randomized mice were treated with control (saline), etravirine (100 mg/kg × three times per week by i.p. injections), paclitaxel (15 mg/kg once a week), and a combination group for three weeks. Representative fluorescence images of female nude mice after three weeks of treatment. (**c**,**d**) Tumor weight and tumor size were measured at the end of the treatment. (**e**) Mice’s body weights were monitored weekly. (**f**,**g**) Metastatic tumors were counted and measured. (**h**) Representative H&E staining, IHC images, and quantification of cell proliferation marker PCNA, AGR2, angiogenesis marker CD31, and apoptosis marker cleaved caspase-3. Data are presented as mean ± SEM. One-way ANOVA and t-test analyses. * *p* < 0.05, ** *p* < 0.01, *** *p* < 0.001.
