# Supplementary material for: Inhibitory Effect of Etravirine, a Non-Nucleoside Reverse Transcriptase Inhibitor, via Anterior Gradient Protein 2 Homolog Degradation against Ovarian Cancer Metastasis
Source: Int J Mol Sci. 2022 Jan 15;23(2):944. doi: 10.3390/ijms23020944 (PMC8777939; doi:10.3390/ijms23020944)

International Journal of
Molecular Sciences

MDPI

Supplementary Figure S1. AGR2 expression in A2780 and A2780-ADR cells.

(a) Western blot analysis showing the AGR2 level. (b) mRNA level of AGR2 in A2780 and A2780-ADR cells. (c) Intracellular staining analysis of AGR2 expression in the monolayer and spheroids of A2780 and A2780-ADR cells. Data are expressed as mean ± SEM. T-test analysis. **p < 0.01.

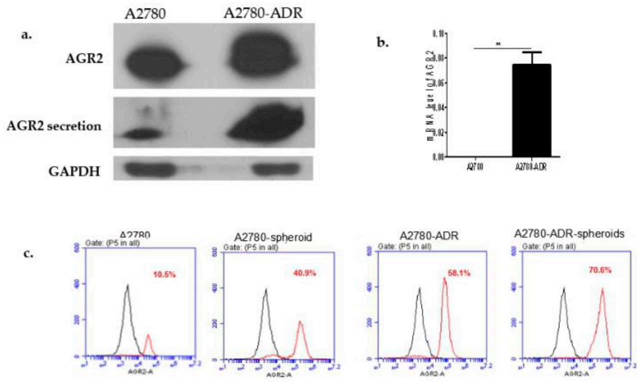

Supplementary Figure S2. Etravirine induce autophagy. (a) A2780-ADR cells were treated with etravirine 5 uM for 72 h and CQ 50 uM for 24 h. The effects of etravirine on autophagy were tested by using Cyto-ID staining. Scale bar = 100 µm. (b) A2780 and A2780-ADR cells were treated with etravirine (up to 10 uM) for 72 h. Western blot analysis showed the effect of etravirine on ULK1 expression.

a.

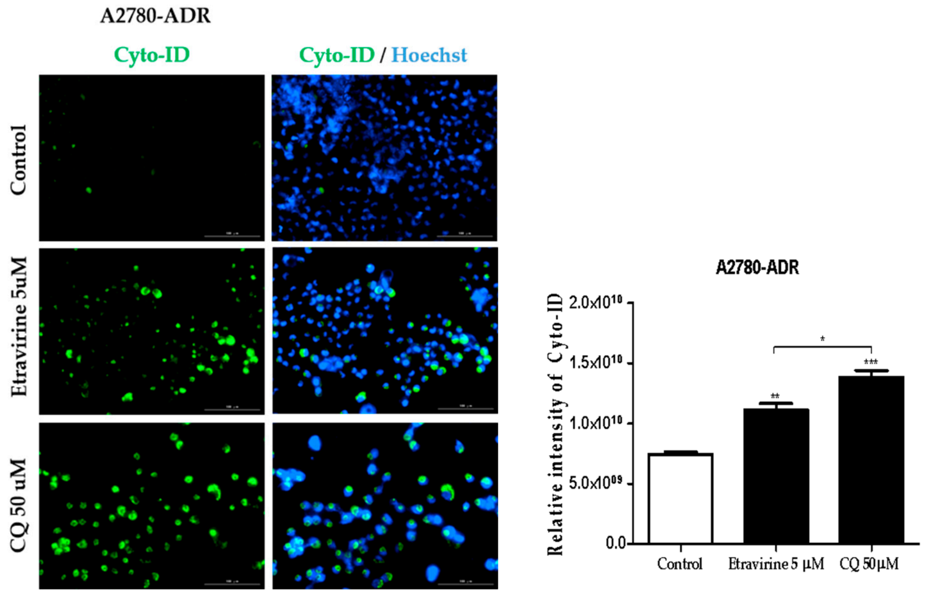

b.

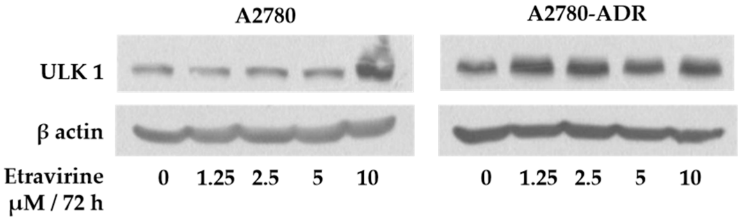

Supplementary Figure S3. Etravirine impair cell viability of SKOV3 and OVCAR8 cells.

SKOV3 and OVCAR8 cells were treated with severals concentrations of etravirine for 72 hours and then cell viability were determined by CCK8 assay (dataare presented as percentages versus the control)

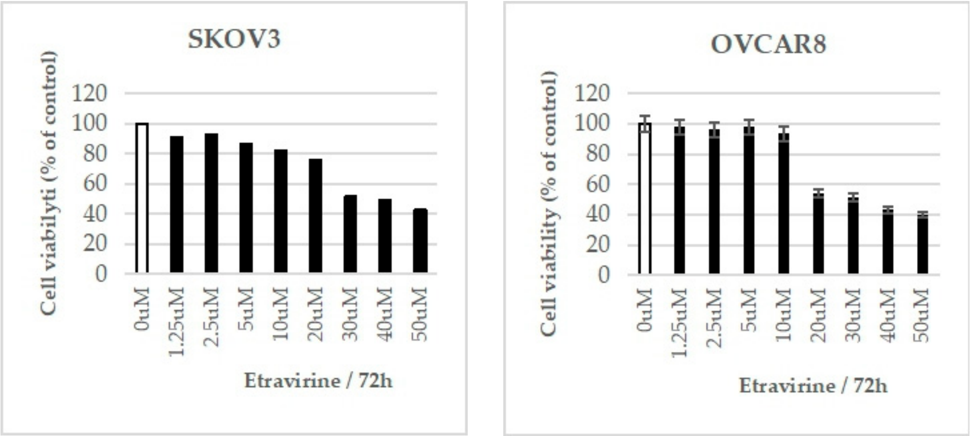

Supplementary Figure S4. Effect of etravirine on colony formation in A2780 and A2780-ADR cells.

(a, b) Etravirine inhibits the colony formation ability of A2780 and A2780-ADR cells

(c, d) Synergistic effect of 5 nM paclitaxel and 5 µM on colony growth in A2780 and A2780-ADR cells

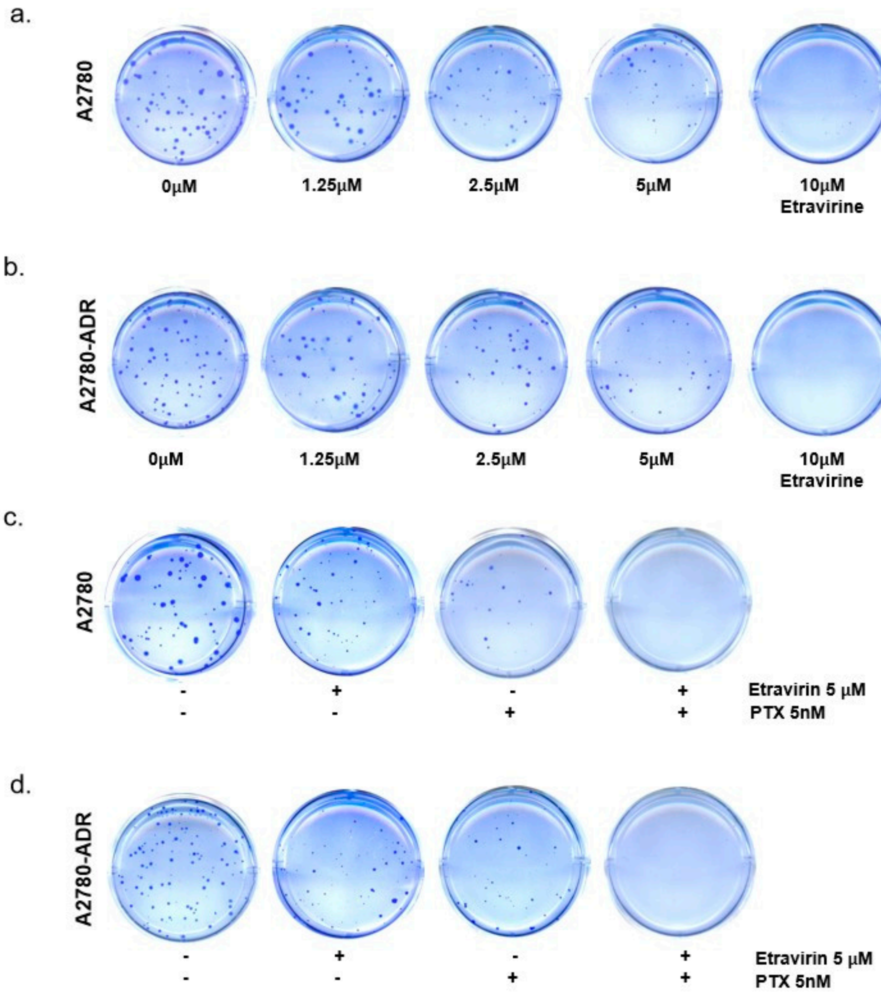

Supplementary Figure S5. Representative H&E staining of the heart, kidneys, lungs, liver, and spleen.

Toxicology studies were conducted at the end of the experiment. The major organs were harvested and stained with H&E. Scale bar = 100 μm.

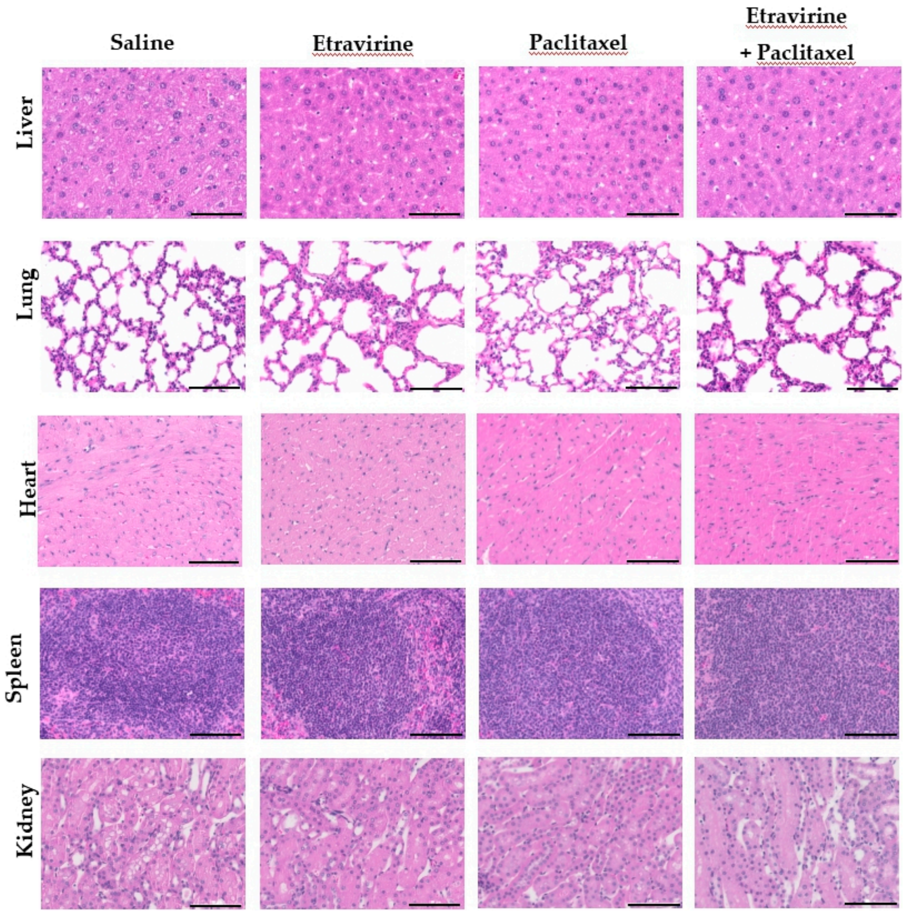

Supplement: Supplementary file 1 [file ijms-23-00944-s001.zip › ijms-1479679-supplementary.pdf]
